# Sexual offending runs in families: A 37-year nationwide study

**DOI:** 10.1093/ije/dyv029

**Published:** 2015-04-06

**Authors:** Niklas Långström, Kelly M Babchishin, Seena Fazel, Paul Lichtenstein, Thomas Frisell

**Affiliations:** ^1^Department of Medical Epidemiology and Biostatistics, Karolinska Institutet, Stockholm, Sweden, ^2^Swedish Prison and Probation Administration, Norrköping, Sweden, ^3^University of Ottawa, Institute of Mental Health, Ottawa, Canada, ^4^University of Oxford, Department of Psychiatry, Oxford, UK and ^5^Clinical Epidemiology Unit, Department of Medicine, Karolinska Institutet, Stockholm, Sweden

**Keywords:** Crime, sexual, violence, rape, sexual abuse, family relations

## Abstract

**Background:** Sexual crime is an important public health concern. The possible causes of sexual aggression, however, remain uncertain.

**Methods:** We examined familial aggregation and the contribution of genetic and environmental factors to sexual crime by linking longitudinal, nationwide Swedish crime and multigenerational family registers. We included all men convicted of any sexual offence (*N = *21 566), specifically rape of an adult (*N = *6131) and child molestation (*N* = 4465), from 1973 to 2009. Sexual crime rates among fathers and brothers of sexual offenders were compared with corresponding rates in fathers and brothers of age-matched population control men without sexual crime convictions. We also modelled the relative influence of genetic and environmental factors to the liability of sexual offending.

**Results:** We found strong familial aggregation of sexual crime [odds ratio (OR) = 5.1, 95% confidence interval (CI) = 4.5–5.9] among full brothers of convicted sexual offenders. Familial aggregation was lower in father-son dyads (OR = 3.7, 95% CI = 3.2–4.4) among paternal half-brothers (OR = 2.1, 95% CI = 1.5–2.9) and maternal half-brothers (OR = 1.7, 95% CI = 1.2–2.4). Statistical modelling of the strength and patterns of familial aggregation suggested that genetic factors (40%) and non-shared environmental factors (58%) explained the liability to offend sexually more than shared environmental influences (2%). Further, genetic effects tended to be weaker for rape of an adult (19%) than for child molestation (46%).

**Conclusions:** We report strong evidence of familial clustering of sexual offending, primarily accounted for by genes rather than shared environmental influences. Future research should possibly test the effectiveness of selective prevention efforts for male first-degree relatives of sexually aggressive individuals, and consider familial risk in sexual violence risk assessment.

**Key Messages**

- Nationwide Swedish data of all 21 566 men convicted of a sexual crime over 1973–2009 and matched controls suggest substantial familial risk of sexual offending.
- Sexual offending is primarily accounted for by genetic and unique environmental risk factors rather than shared environmental influences.
- Selective prevention efforts may be indicated for male first-degree relatives of sexually violent men; and, among at-risk individuals, taking into account a family history of sexual offending might improve the prediction of sexual violence.

## Introduction

Sexual aggression is a substantial social and public health problem;^1–3^ approximately one-quarter of women and one-tenth of men report being sexually victimized in their lifetime.^4–8^ Sexual abuse experiences are associated with a wide range of negative physical, sexual and mental health outcomes.^9^ Currently, prevention efforts are mostly focused on relapse prevention among individuals who have already committed a sexual offence,^10^ but three recent systematic reviews failed to find high quality evidence for the effectiveness of existing sex offender treatment programmes.^10–12^ This highlights the need for renewed efforts to identify causal risk factors of sexual aggression, as such determinants might yield more promising targets for intervention.

Alongside intergenerational transmission of violent outcomes in general,^13–16^ typically interpreted to suggest environmentally mediated mechanisms, there is support that childhood sexual victimization might increase adult risk of sexual offending.^17–20^ This is one possible pathway, but little is known about others. Two small studies have examined the intergenerational transmission of paedophilia^21^^,^^22^ and another, more generally, sexual interest in youth under age 16.^23^ In the latter investigation, a large twin sample was used and found that unique (non-shared) environment rather than genetic factors contributed the most to variability in sexual interest in youth and associated masturbatory fantasies,^23^ suggesting that paedophilic sexual interest was not aggregated within twin sets.

The patterns of possible familial aggregation of sexual offending could inform the extent to which sexual offending is accounted for by genetic, non-shared and shared (common) environmental influences. We used a Swedish total population sample with longitudinal register data for all convicted male sexual offenders (*N = *21 566) over 37 years. Familial risks were described for sexual offending across different levels of relatedness and two sexual offence subtypes. We hypothesized a moderate to strong genetic influence and small shared family environment effects on sexual offending, as earlier studies indicated strong genetic influence for the related domains of non-sexual violence,^24^^,^^25^ sexual orientation,^26^ problematic sexual behaviour in children^27^and sexual dysfunction.^28^

## Methods

### Dataset and variables

We linked several Swedish total population registers using the unique personal identification number as key. From the Crime Register (held by the National Council of Crime Prevention), we obtained records of all convictions in Swedish general courts between 1 January 1973 and 31 December 2009. The Multi-Generation Register (Statistics Sweden) identified the biological parents of everyone living in Sweden at any time since 1961 (including those who immigrated to Sweden as children together with their parents). This information made it possible to link full and half-siblings, and to construct family pedigrees to analyse familial aggregation at several levels of genetic and family environmental distance. Through this linkage, we identified a total of 11 931 785 individuals and 21 566 convicted male sexual offenders. Since less than 1% of convicted sexual offenders were female, only male offenders and their fathers and brothers were included in the sample.

For each sex offender, we drew five controls without history of sexual offending matched on the age of both the offender and his father or brother (depending on the studied relationship). We calculated conditional odds ratios for the risk of sexual offending in fathers and brothers of probands (i.e. sexual offenders) compared with corresponding rates in the relatives of controls. The Total Population Register (Statistics Sweden) provided information on individuals’ sex and birth year. We used data from the Cause of Death- and Migration Registers (both at Statistics Sweden) to compute individual time-at-risk; that is, when study subjects were alive and living in Sweden.

We defined sexual crime as a conviction for any sexual offence according to the Swedish Penal Code. This included the three main categories: (i) rape and sexual coercion against an adult; (ii) intra- and extra-familial child molestation or child rape (under age 15 years or, if the offending adult has a position of authority, under the age of 18), which (motivated by the connection to paedophilia) also included possession/distribution of child pornography; and (iii) non-contact sexual offences such as sexual harassment, indecent exposure or exhibitionistic acts. Attempted and aggravated offences were included whenever applicable. Non-sexual violence was defined as a conviction for any non-sexual violence offence according to the Swedish Penal Code, such as homicide, (aggravated) assault, (aggravated) robbery or (aggravated) illegal threats (see ^14^ for details). Plea-bargaining is forbidden in the Swedish judicial system and all crimes are registered regardless of possible offender insanity at the time of perpetration. Hence, the register includes individuals who suffered from psychosis at the time of the offence (usually referred to compulsory inpatient forensic psychiatric care). Further, conviction data include all persons who receive custodial or non-custodial sentences in court as well as cases where the prosecutor decided to caution or fine. Finally, Sweden does not differ considerably from other members of the European Union regarding rates of violent crime and their resolution.^29^

### Analyses

For each studied degree of relatedness (son-father, full brother, maternal half-brother and paternal half-brother), we first created a dataset containing all such relatives of each male individual from the Multi-Generation Register. That is, one entry per index person-relative pair rather than one entry per individual. Second, we performed a nested case-control study with multiple matching variables. Hence, when a person was convicted of a sexual crime, he was considered a case and five controls were randomly chosen among people who were alive, living in Sweden and not convicted of a sexual crime at the time of the case’s conviction. Controls were matched to cases on sex, birth year and having a corresponding relative (e.g. father or brother, respectively) of the same age. If a father or brother of the index person had ever been convicted of a sexual crime during the 37-year study period, the index person was considered exposed.

We analysed the difference in exposure between cases (index offenders) and controls using conditional logistic regression with a robust sandwich estimator, yielding odds ratios (ORs) and 95% confidence intervals (95% CIs). The sandwich estimator aggregated over families (e.g. among sibling pairs having the same parents) to adjust for the correlated nature of family data. This analysis was performed for index offenders’ biological fathers and full and half-brothers, respectively. Finally, recognizing that our definition of sexual crime contained several, perhaps aetiologically disparate, subtypes, we also stratified analyses according to rape against an adult and child molestation sexual offender subtypes. No further stratifications were done to avoid impaired statistical power. All calculations were performed using proc phreg in SAS v. 9.2 and the study was approved by the Regional Ethics Committee in Stockholm (decision reference number 2009/939-31/5).

### Quantitative genetics

The heritability of sexual offending was estimated based on the familial risks among full and half-brothers using a generalized linear mixed model (GLMM) with a probit link.^30^ Models were adjusted for the difference in base prevalence of sexual offending between half-brothers and full brothers and for birth decade. GLMM yields similar results as structural equation models, and has been described in more detail in previous applications to post-term delivery,^31^ the comorbidity of bipolar disorder and schizophrenia,^32^ and non-sexual violent offending.^24^

Briefly, the probit link models the binary outcome as coming from a standard normal distribution with a distinct threshold. Whereas everyone is assumed to have a value on this underlying liability, only those with values above the threshold are convicted of a sexual crime. The liability value is presumably a sum of genetic and environmental contributions. Since familial aggregation of sexual offending can be described as covariation of the family members’ liability values for this crime, and family members have known genetic relatedness, we used the correlations between relatives at different genetic and environmental distances to estimate the relative contributions of genetic and environmental factors to the liability of sexual offending. Under random mating, the additive genetic correlation is 0.50 for full brothers and 0.25 for half-brothers.

Family environment was assumed to be shared (i.e. perfectly correlated) among full siblings and maternal half-siblings. In contrast, we assumed it to be non-shared (i.e. uncorrelated) by paternal half-brothers. A previous study with a related sample of violent non-sexual offenders found that 83% of maternal half-siblings were indeed registered as living in the same home compared with only 3% of paternal half-siblings.^24^ Under assumptions of minimal or no statistical interaction or correlation between genetic and environmental factors, and no direct effects of one relative’s phenotype (sexually violent offending or not) on the other’s, expected correlations can be used to predict observed phenotypic correlations, hereby providing estimates for the proportion of sexual crime convictions that are due to genetic and shared environmental factors. The residual variance in liability is often described as due to ‘unique environment’, and is not explicitly estimated in the GLMM.

## Results

### Sample characteristics

As in other countries, rates of sexual offending and the demographic composition of the Swedish population change over time. Importantly, possible effects of these changes on familial risk estimates were accounted for by matching in a nested case-control design. The dynamic nature of the cohort made it difficult to summarize individual characteristics for the full sample. Instead, we present descriptive information for a restricted cohort of men representing about a quarter of the full sexual offender sample; 5028 male sex offenders who were alive, living in Sweden and 30–45 years old in 2009 (see Table 1); 46 women representing 0.90% of the cohort were excluded. Male sex offenders comprised all men convicted of rape of an adult (*n = *1577; 30.7%), child molestation (*n = *961; 19.1%), and other sexual offences (e.g. exhibitionism; *n* = 2523; 50.2%). A total of 92 offenders had separate convictions for adult rape and child molestation (*n = *92; 1.8%) and were counted in both these categories. A substantial proportion of sex offenders were also convicted of non-sexual violent offences (46.2%; *n* = 2323); men convicted of rape of an adult more often had also one or more non-sexual violent convictions (64.4%; *n = *995) than those convicted of child molestation (30.1%; *n = *289).

**Table 1.** Sociodemographic characteristics for all men who were alive, living in Sweden, and 30 to 45 years old in 2009 (*N* = 1 027 139)

Characteristic	*N*
Childhood socioeconomic position, *n* (%)	
Low	309 423 (30.1)
Medium	286 773 (27.9)
High	211 075 (20.6)
Missing	219 868 (21.4)
Country of birth, *n* (%)	
Sweden	830 349 (80.8)
Other Scandinavian country	22 750 (2.2)
Non-Scandinavian country	174 040 (16.9)
Criminal conviction, *n* (%)	
Any non-sexual violent crime	73 760 (7.2)
Any sexual crime	5 028 (0.5)
Rape	1 544 (0.2)
Child molestation	961 (0.1)
Non-contact sexual offence^a^	2 523 (0.2)
Age at first sexual offence, mean (SD)	28.5 (7.7)

^a^Non-contact sexual offences included crimes such as sexual harassment, indecent exposure and exhibitionistic acts.

### Familial risk of any sexual offending

We found substantial familial aggregation of sexual violence leading to a criminal conviction among men (see Table 2). Overall, first-degree biological relatives living in the same family, i.e. full brothers and son-father dyads, had the highest familial risk (Table 2). For instance, the odds ratio (OR) for any sexual offending was 5.1 (95% CI = 4.5–5.9) among full brothers of sexual offenders compared with the age-matched brothers of individually age-matched control individuals. Since familial aggregation cannot in itself disentangle genetic from environmental effects, we explored this issue further by computing heritability estimates reported below.

**Table 2.** Relative risk of sexual violence convictions among fathers and brothers of all 21 566 men convicted of any sexually violent crime in the Swedish total population 1973–2009, compared with fathers and brothers of controls without a conviction for sexual offences

Relative			Father or brother’s sexual conviction type					
Relation to index male	No. of dyads	No. of affected index males	Any sexual crime (*n* = 21 566)		Rape of an adult (*n* = 6131)		Child molestation (*n* = 4465)	
			No. of concordant pairs	Matched OR (95% CI)	No. of concordant pairs	Matched OR (95% CI)	No. of concordant pairs	Matched OR (95% CI)
Father	3 698 623	13 991	200	**3.7 (3.2–4.4)**	53	**3.1 (2.3–4.2)**	59	**4.3 (3.2–5.9)**
Brother	3 103 618	13 420	392	**5.1 (4.5–5.9)**	153	**6.2 (5.0–7.8)**	84	**5.9 (4.5–7.8)**
Paternal half-brother	378 948	3 026	56	**2.1 (1.5–2.9)**	14	1.7 (1.0–3.1)	15	**2.3 (1.3–4.0)**
Maternal half-brother	348 026	3 037	58	**1.7 (1.2–2.4)**	22	1.7 (1.0–2.7)	16	**4.1 (2.3–7.4)**

Sexual violence conviction rates among fathers and brothers of index men convicted of sexual offences were compared with rates among corresponding relatives of control men (non-convicted of sexual offence) matched on birth year of index male and his father/brother. Each individual may appear in different categories (e.g. father, brother) depending on family pedigree. The numbers of concordant pairs in the rape of an adult and child molestation offence types do not add up to that in the any sexual crime category. This occurred since other sexual offences, primarily non-contact and harassment types, were excluded from subgroup analyses. Figures in bold reached *P* < 0.05.

### Familial effects by sexual offence subtype

**Figure 1.**
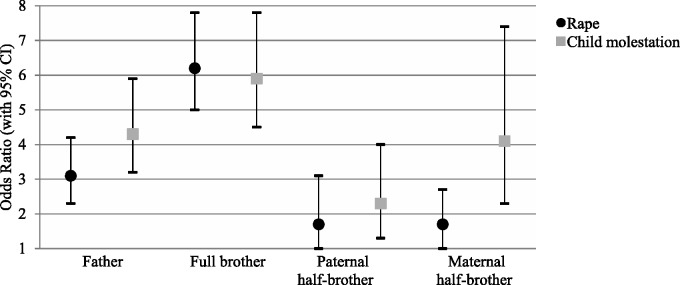
Relative risk of sexual crime among first-degree male relatives and half-brothers of men convicted of sexual offences in the Swedish total population 1973–2009, compared with male relatives of matched controls. Points represent odds ratios (ORs) and bars their 95% confidence intervals (CIs) for rape and child molestation.

‘Any sexual crime’ included offence subtypes that might represent diverse aetiologies. For instance, this may be through more general criminogenic vulnerabilities (such as impulsivity and antisocial cognitions) being more pronounced in rape of an adult, and paedophilic disorder in child molestation. Hence, we specifically analysed full brothers’ risk (because of the highest statistical power) separately for rape of an adult and child molestation, respectively (Figure 1). First, when a brother had been convicted of any sexual crime, the risk was not differently increased for his brother’s conviction of any sexual offence (OR = 5.1, 95% CI: 4.5–5.9) compared with rape of an adult (OR = 6.2, 5.0–7.8) and child molestation (OR = 5.9, 4.5–7.8; Table 2). Second, however, specificity of familial aggregation by sexual offence subtypes was suggested since the familial aggregation among full brothers of the rape of an adult was higher (OR = 17.4, 11.9–25.4) than for child molestation (OR = 7.7, 4.8–12.3; Table 3).

**Table 3.** Relative risk of rape and child sexual molestation convictions among brothers of sexually offending male probands (index offenders) in the Swedish total population 1973–2009

No. of dyads	No. of affected index males	Brother’s sexual conviction type			
		Rape of an adult		Child molestation	
		No. of concordant pairs	Matched OR (95% CI)	No. of concordant pairs	Matched OR (95% CI)
**Index brother committed rape of an adult (*n* = 6131)**					
3 103 618	3761	84	**17.4 (11.9–25.4)**	26	**5.4 (3.4–8.5)**
**Index brother committed child molestation (*n* = 4465)**					
3 103 618	2696	26	**7.3 (4.4–12.2)**	34	**7.7 (4.8–12.3)**

Figures in bold reached *P* < 0.05. Rates of convictions among brothers of convicted index males were compared with rates among brothers of control men matched on birth year of index male and his brother(s).

### Heritability estimates

Using a probit GLMM with full and half-brothers, we estimated that genetic factors contributed 40% (95% CI = 17%–48%) of the variability in any sexual crime compared with 2% (95% CI = 0%–13%) by the shared family environment. Shared environment would include factors that are primarily constant across children growing up together, such as parental attitudes and neighbourhood. The remaining variance (58%) was explained by unique environment (e.g. perinatal adversities, biological factors, and social events and processes) not shared by brothers, and measurement error. Although point estimates suggested greater heritability for child molestation than for rape, the statistical power was limited and confidence intervals overlapped. Specifically, genetic factors tended to explain less variance in rape of an adult (heritability = 19%, 95% CI = 0%–57%; shared environment = 15%, 95% CI = 0%–29%; non-shared, unique environment and measurement error = 66%) than for child molestation (heritability = 46%, 95% CI = 0%–59%; shared environment = 0%, 95% CI = 0%–29%; non-shared, unique environment and measurement error = 54%).

## Discussion

We addressed familial aggregation of sexual offending by comparing relatives of 21 556 male sexual offenders with relatives of matched non-offender controls. This study is also the first, to our knowledge, that estimates the relative influence of genetic and environmental factors on the development of serious sexually aggressive behaviour. We found substantial evidence of moderate to strong excess familial risk for sexual offending among men. Having a father or a brother convicted of a sexual offence increased the odds of being convicted oneself 4 to 5 times compared with age-matched control men without a sexually aggressive father or brother. These familial aggregation effects are comparatively large in relation to familial risks for other studied behaviours, including odds ratios for violent crime of about 3.5 in children of male violent offenders^14^ and about 2 for suicidal behaviour in children of individuals who completed suicide.^33^ Further, although comparisons with medical conditions are quite different as sexual offending is not a disease, Frisell *et al*. reported an odds ratio of about 3 for diagnosed rheumatoid arthritis, a disorder with similar population prevalence as sexual offending, in first-degree relatives with this disease.^34^ In keeping with our hypothesis, aggregation patterns suggested that genes rather than shared environmental factors explained familial aggregation. Specifically, statistical modelling indicated that sexual offending was primarily influenced by genes (40%) and non-shared environmental factors, including measurement error (58%).

Our findings may inform selective, or secondary, public health approaches to reduce sexual violence. First, first-degree family members of sexual offenders, although very unlikely to be responsible for the sexual aggression of the offender, may benefit from interventions heightening their risk and sexual boundary awareness, and improving their communication and conflict management skills. Second, male first-degree relatives of sexual offenders could specifically be offered psychological and pharmacological help to decrease individual risk factors such as cognitive distortions, emotional instability and hypersexuality. Both these intervention options are likely better tolerated, and may also reduce other adverse outcomes among family members (e.g. non-sexual crime, substance misuse) if integrated in family interventions targeting general risk factors. Since selective prevention strategies are relatively new to the field of sexual offending in contrast to universal (primary) and indicated (tertiary) prevention efforts (treatment) for known sexual offenders,^10^^,^^11^ development of such prevention efforts should be carefully monitored and evaluated.

The present findings might also guide attempts to improve the assessment of sexual violence risk. Current instruments for such assessments do not include risk items related to family history;^35^ hence, more work is needed to establish if including familial history of sexual aggression improves predictive accuracy with persons at risk. Both prevention and risk prediction efforts, however, should consider the low absolute base rates of sexual offending, also in family members of sexual offenders (e.g. 2.5% convictions in full brothers), that will for example result in modest positive predictive values.^36^ With such likely positive predictive values, any interventions offered to family members should avoid harm.^37^

There are a number of limitations with this study. It is estimated that up to 80% of all sexually abusive acts are never reported to the police.^4^^,^^6^ Additionally, when sexually abusive experiences are reported, many do not result in charges or convictions.^38^ Hence, familial risk estimates could, at least partly, reflect not only the liability to commit a sexual offence but also characteristics that increase the probability of being arrested and convicted. Conversely, conviction data represent the more severe end of the offending spectrum, are not affected by recall bias and other informant biases inherent with self-report data, and allow for international comparisons of our findings. Further, despite using a complete national sample of sexual offenders over almost four decades, the study’s statistical power was limited. Hence, in particular, estimates of familial aggregation and heritability for rapist and child molester subgroups should be interpreted with caution. For example, both the trend towards stronger association of child molestation convictions in maternal compared with paternal half-siblings (Table 2), suggesting a shared environmental influence, and the heritability estimates for child molestation compared with rape of an adult, had overlapping confidence intervals. Further, potential differences between sexual offender subgroups may have been attenuated by the inclusion of offenders without prepubescent victims (the latter more strongly suggesting paedophilia) in the child molester category. Sweden, like many other countries, legally defines children as individuals under the age of 15; or, if the offending adult had a position of trust, under age 18 years. Also, caution is needed regarding generalization of the relative importance of genetic and non-shared environmental influences on sexual offending to countries and settings with poorer gender equality and sexual rights policies. Finally, although large enough samples may be difficult to obtain, twin or adoption designs would be useful in the further study of the intergenerational transmission of sexual offending.

In conclusion, we found substantial familial aggregation of male sexual offending. Having a father or a brother convicted of a sexual offence increased the odds of being convicted in a particular man 4–5-fold compared with men without a sexually aggressive father or brother. Statistical modelling of the overall liability to sexual aggression based on the strength and pattern of familial aggregation suggested that genetic and non-shared environmental factors were more important than shared environmental influences. Further, genetic effects tended to be stronger for child molestation than for rape. The findings might inform aetiological theories of sexual offending, the development of targeted, selective prevention programmes for at-risk families, and applied risk assessment.
